# Assessment of the Effectiveness of Rehabilitation after Total Knee Replacement Surgery Using Sample Entropy and Classical Measures of Body Balance

**DOI:** 10.3390/e23020164

**Published:** 2021-01-29

**Authors:** Anna Hadamus, Dariusz Białoszewski, Michalina Błażkiewicz, Aleksandra J. Kowalska, Edyta Urbaniak, Kamil T. Wydra, Karolina Wiaderna, Rafał Boratyński, Agnieszka Kobza, Wojciech Marczyński

**Affiliations:** 1 Department of Rehabilitation, Faculty of Medical Sciences, Medical University of Warsaw, 02-091 Warsaw, Poland; anna.hadamus@wum.edu.pl (A.H.); dariusz.bialoszewski@wum.edu.pl (D.B.); karolina.wiaderna@wum.edu.pl (K.W.); 2 The Józef Piłsudski University of Physical Education in Warsaw, 00-809 Warsaw, Poland; 3 Professor Adam Gruca Independent Public Teaching Hospital in Otwock, Rehabilitation Clinic, 05-400 Otwock, Poland; aleksandra.macheta@wp.pl (A.J.K.); edyta.urbaniak@wp.pl (E.U.); kamil.wydra@interia.eu (K.T.W.); borek14@interia.eu (R.B.); akobza@poczta.fm (A.K.); 4 Medical Centre for Postgraduate Education, Professor Adam Gruca Independent Public Teaching Hospital in Otwock, Orthopaedics Clinic, 05-400 Otwock, Poland; wmarczynski@interia.pl

**Keywords:** total knee replacement surgery, knee arthroplasty, osteoarthritis, virtual reality, sample entropy, body balance

## Abstract

Exercises in virtual reality (VR) have recently become a popular form of rehabilitation and are reported to be more effective than a standard rehabilitation protocol alone. The aim of this study was to assess the efficacy of adjunct VR training in improving postural control in patients after total knee replacement surgery (TKR). Forty-two patients within 7–14 days of TKR were enrolled and divided into a VR group and a control group (C). The C group underwent standard postoperative rehabilitation. The VR group additionally attended twelve 30-min exercise sessions using the Virtual Balance Clinic prototype system. Balance was assessed on the AMTI plate in bipedal standing with and without visual feedback before and after the four-week rehabilitation. Linear measures and sample entropy of CoP data were analyzed. After four weeks of rehabilitation, a significant reduction in parameters in the sagittal plane and ellipse area was noted while the eyes remained open. Regression analysis showed that sample entropy depended on sex, body weight, visual feedback and age. Based on the sample entropy results, it was concluded that the complexity of the body reaction had not improved. The standing-with-eyes-closed test activates automatic balance mechanisms and offers better possibilities as a diagnostic tool.

## 1. Introduction

The complex interaction of somatosensory, visual and vestibular feedback networks, numerous brain regions, and the musculoskeletal system creates a system involved in postural control [1]. Postural control is a term used to describe the way in which the central nervous system (CNS) regulates information from other systems to produce appropriate motor output to maintain an upright and controlled posture. It has been demonstrated [2,3,4] that ageing impairs the capability of the CNS to process these signals and reduces the capacity to modify adaptative reflexes. Additionally, damage to any of these balance regulation levels influences the output of the postural system, resulting in an increased risk of postural instability, falls and consequently fractures.

Osteoarthritis is the most prevalent rheumatic joint disorder that results from breakdown of joint cartilage and underlying bone [5]. Osteoarthritis affects mostly older people, causing joint pain, stiffness, and swelling; changes in the way the joint moves; and a feeling that the joint is loose or unstable. Treatment of osteoarthritis is a multistage process involving multidisciplinary care [6,7,8]. The underlying principle is to limit the risk factors or, in later life, factors that accelerate the degenerative process. Surgery is considered in cases of advanced disease that significantly limits daily activity, is associated with difficult-to-control pain and does not respond to other treatment modalities. In osteoarthritis of the knee or hip joint, the affected joint is most commonly replaced with an endoprosthesis during an arthroplasty procedure. It is expected that a patient with long-standing disease and following a surgical intervention may experience significant problems maintaining balance and walking due to damage to neighboring tissues, such as bone, muscle, ligaments, tendons and skin. Accordingly, postoperative rehabilitation is indispensable in ensuring successful surgical outcomes. Basically, rehabilitation programs in surgical OA patients include range of motion exercises, muscle strengthening exercises, improvements in function of the operated joint (including proprioception), and physical therapy procedures to relieve swelling, inflammation and pain and improve healing processes [9]. The rehabilitation program can be modified to achieve optimal therapeutic outcomes depending on the specific indication for surgery, surgical procedures and patient characteristics (age, overall mental and physical health, and preoperative status). Such modifications include virtual reality (VR) technology-based training.

Virtual reality (VR) games use interactive computer environments that appear to be real to improve daily activities or functional movements [7]. The patient is able to interact with a virtual environment using specific devices or body movements. Games may provide an avatar that represents the patient’s body movements [7]. Numerous publications report that exercises in VR can increase patient motivation, especially in children and the elderly, and help to document their progress [7,10,11,12]. Additionally, VR games can be used as an affordable tool for home exercise in older adults [11,13]. VR games are mostly used in restoring body balance and gait functions [11,14] and, less often, to improve other functions, such as muscle strength [11]. Despite promising effects, the quality of scientific evidence supporting the use of VR is insufficient to recommend using it in everyday rehabilitation routines, especially in patients with orthopedic conditions or dysfunctions [7].

Diverse indicators have been explored in postural stability assessments, mostly based on the trajectory of the center of pressure (CoP). Classical approaches focus on CoP variability based on the notion that it displays extensive irregularities and nonstationary fluctuations during quiet standing. Therefore, linear tools, such as the CoP path length, sway velocity and area, quantify the amount of CoP movement during a specific task, independently of their order in the distribution. To better understand the physiology of postural control, nonlinear measures should be applied [15,16]. The nonlinear system approach helps evaluate different aspects of the CoP data. Nonlinear measures are able to capture the temporal component of the movement variation in CoP with regards to how motor behavior develops over time. These measures make it possible to quantify the regularity, complexity, and efficiency or ‘automaticity’ of postural control [15,16,17]. Nonlinear tools include the largest Lyapunov exponent and Hurst exponent, fractal dimension and entropy families [15,16,18].

The entropy family quantifies the regularity of a signal with predictable (for example periodic) signals resulting in low entropy and completely unpredictable signals resulting in high entropy. In addition to multiscale entropy (univariate or multivariate) and approximate entropy, sample entropy is one of the nonlinear parameters often used to calculate from CoP data. In contrast to approximate entropy, sample entropy does not depend on record length and is characterized by relative consistency [19]. Sample entropy has been used to analyze CoP data in stationary patients [19,20,21,22] as well as during gait [23,24] and different physical activities or movements [25,26]. A decrease in sample entropy, which means more regular sway fluctuations, is interpreted as more rigid postural behavior and consequently a decrease in the effectiveness of postural control [19,25]. More rigid postural behavior results in fixed balance control patterns and consequently dysfunctional balance control during perturbations [21,27]. Comparing entropy-based variables to classic variables, entropy measurements provide information about the quality of sway related to task difficulty, whereas classic variables quantify the amount of CoP movement during a specific task or the amount of variation present in a set of values, independent of their order in the distribution measure [28]. Moreover, increased values of sample entropy, which indicate greater irregularity in the CoP value, may be attributed to a reduced amount of attention invested in posture [16] and may be interpreted as an increase in the ”automaticity” of postural control [15].

The objective of the study was to assess the effect of adjunct VR-based training on changes in balance parameters, including classic measures and sample entropy, in the short period after knee arthroplasty.

## 2. Materials and Methods

### 2.1. Participants

The study enrolled 42 patients within 7–14 days after total knee replacement surgery (TKR). All patients were operated on at the Orthopedic Department of Professor Adam Gruca Independent Public Teaching Hospital in Otwock. The inclusion criteria comprised (1) noncomplicated total knee replacement surgery because of primary knee osteoarthritis, (2) no other balance problems (due to neurological or heart diseases, vertigo, etc.), (3) no current musculoskeletal complaints other than related to the operated joint, and (4) written consent to participate in the study.

Patients meeting the inclusion criteria were divided using a block randomization technique [29] into two random groups, including a study (VR) group and a control (C) group. Each group consisted of 21 patients (14 women, 7 men) whose characteristics are presented in Table 1.

### 2.2. Procedures

All patients qualified for the study attended a standard four-week protocol of stationary rehabilitation involving five rehabilitation sessions per week with each session lasting approximately 4 h. Rehabilitation procedures were performed by physiotherapists (A.K. and R.B.). Treatment included individual exercises (to increase knee range of motion, muscle strengthening and stretching), continuous passive motion exercises, gait and balance exercises, manual therapy and massage (with a focus on soft tissue and patellar and scar mobilization), cryotherapy in the operated area, laser therapy for scarring, magnetic field therapy and kinesiology taping applications.

The study group additionally received 12 sessions (three sessions per week) of virtual reality games on the Virtual Balance Clinic (VBC) prototype system (VBC-Project Consortium, Warsaw, Poland). The VR games were applied concurrently with other treatments. The VBC system consists of a balance plate and a “Kinect 2” camera. The balance plate allows the measurement of displacement of the center of pressure (CoP) in real time, and the “Kinect 2” camera is used to trace body movements. Accordingly, VBC software allows for qualifying each movement performed during exercises as “correct” or “false” or quantifying some movements as “partly correct”. The VBC system offers the choice of nine different games, including tasks, such as CoP movement in the sagittal or frontal plane or diagonally during standing with both legs parallel or toe to heel standing, maintaining balance during upper body movements (such as trunk rotations and/or arm movements), one leg standing, forward or side steps, or walking in place. Each session lasted 30 min and included three different games, and each patient played all games for the same length of time during the rehabilitation period. The level of difficulty was adjusted individually for each patient by a physiotherapist supervising the exercises. Each patient was assessed twice: before and after the four-week rehabilitation. All patients completed their rehabilitation protocol.

The study protocol was approved by the Bioethics Committee of the Medical University of Warsaw (no. KB/28/2014). The study was conducted according to the ethical guidelines and principles of the Declaration of Helsinki.

### 2.3. Body Balance Measurement and Calculation of Entropy

The postural stability data for each subject were recorded using an AMTI AccuSway (Advanced Mechanical Technology Inc., Watertown, MA, USA) plate with Balance Clinic software. Sample rate was set at 100 Hz. Each person completed three trials of bipedal standing with eyes open and three trials of bipedal standing with eyes closed. Each trial lasted 30 s with a one-minute rest between trials. We used the most popular parameters obtained with traditional linear and nonlinear methods, as proposed by various authors [15,16,30]. The linear parameters comprised the range of CoP displacement in the sagittal and frontal planes, CoP path length, CoP velocity and ellipse of 95% confidence. Moreover, sample entropy (SampEn) was calculated for both CoP components, i.e., medio-lateral (ML) and anterior-posterior (AP) sway. SampEn is the negative natural logarithm of the conditional probability that a dataset of length N, having repeated itself within a tolerance r for m points, will also repeat itself for *m* + 1 points without allowing self-matches: $SampEn\left(m,r,N\right)=-ln\left(\frac{A^{m}\left(r\right)}{B^{m}\left(r\right)}\right)$. *B* represents the total number of matches of length *m*, whereas *A* represents the subset of *B* that also matches for *m* + 1. SampEn was calculated using MATLAB (MathWorks, Natick, MA, USA) codes obtained from the Physionet tool [31] with “default” parameter values of *m* = 2 and *r* = 0.2*(standard deviation of the data).

### 2.4. Statistical Analysis

Statistical analysis was performed using Statistica v. 13.1 (TIBCO Software, Inc., Palo Alto, CA, USA), and the cut-off *p*-value was set at 0.05. The results of the patients’ second trials were analyzed. The second trial results were used because the patients would not always comply with the requirements on their first attempt and often reported fatigue on the third attempt. The Shapiro-Wilk test was used to assess the normality of all data distributions. Wilcoxon’s paired rank test was used to compare the effect of rehabilitation on the parameters of postural stability in each group. The study and control groups were subsequently compared using the Mann-Whitney U test to detect significant differences. In the last step, multi-variation analysis of regression using GRETL-GNU Regression, Econometric and Time-series Library version 2019a (Free Software Foundation, Boston, MA, USA) was performed. The regression analysis was performed using the least squares method. The choice of the best model was based on the Akaike information criterion.

## 3. Results

The results section is divided into three subsections. The first subchapter examines differences between the groups and the impact of rehabilitation protocols on postural control. The following section focuses on the visual feedback influence on the postural stability parameters. The last subchapter focuses on regression analysis.

### 3.1. The Impact of Rehabilitation on Postural Control and Group Differences

In the study group, which received additional VR-assisted rehabilitation with the VBC system, all linear parameters decreased in standing with eyes open and eyes closed (Table 2). Additionally, the range of sagittal sway and area of the ellipse decreased while the eyes remained open. The results in the control group were the opposite of those in tests both with eyes open and closed. All linear parameters exhibited increased values after rehabilitation, but the differences were not statistically significant.

SampEn values in the study group decreased in the frontal plane and increased in the sagittal plane in both the closed- and open-eye tests. The same pattern was observed in the control group but only during standing with eyes closed. All these changes were not statistically significant.

A comparison of the study and control groups failed to reveal statistically significant differences in the key anthropometric parameters of the subjects. Similarly, no significant differences were noted between the groups in the Mann-Whitney U test results with regard to the linear and nonlinear parameters and the influence of rehabilitation or visual feedback.

### 3.2. Impact of Visual Feedback in Both Groups

Analysis of the impact of visual feedback on the postural control parameters revealed much greater differences both before and after rehabilitation than those described in the previous section. In both groups before rehabilitation, closing eyes produced non-significantly higher values of all linear and nonlinear parameters. Additionally, the study group registered significantly higher values of SampEn for medio-lateral sway (*p* = 0.006) and path length (*p* = 0.001), whereas the control group demonstrated a significantly (*p* = 0.001) higher value of path length in the measurement with eyes closed.

Testing after the 4-week rehabilitation again revealed increased values of all parameters in standing with eyes closed. The study group registered significantly higher values of path length (*p* = 0.001), ellipse area (*p* = 0.017) and range of AP sway (*p* = 0.0301), whereas the control group additionally demonstrated higher SampEn for AP sway (*p* = 0.019) and ML sway (*p* = 0.014).

### 3.3. Multiple Regression Analysis

**Figure 1:**
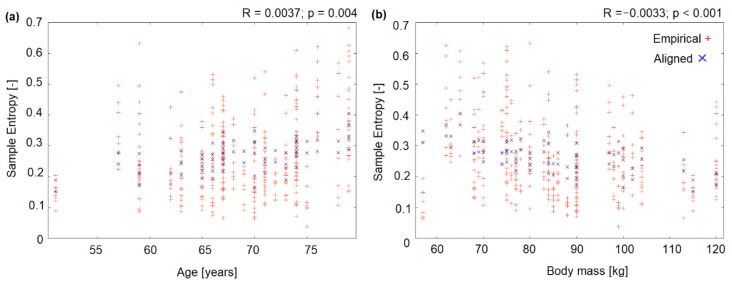
Regression analysis results: (**a**) Empirical (from experiment) and aligned (from model) values of sample entropy versus age, (**b**) Empirical (from experiment) and aligned (from model) values of sample entropy versus body mass.

Regression analysis found that sample entropy depended on sex (with higher entropy values in men; R = 0.0483; *p* = 0.036), body mass (R = −0.0033; *p* < 0.001) (Figure 1a), visual feedback (eyes open vs. closed, with higher entropy in standing with eyes closed; R = 0.0371; *p* = 0.004) and age (R = 0.0037; *p* = 0.004) (Figure 1b). No significant relationship was found for the direction of CoP sway (AP vs. ML), being a member of the study vs. control group, time of assessment (before vs. after rehabilitation), or anthropometric parameters (height or BMI).

## 4. Discussion

This study aimed to assess the effect of adjunct VR-based training on balance parameters in patients undergoing rehabilitation shortly after total knee replacement surgery. Our results indicate that, unfortunately, no significant improvement in the postural stability parameters assessed was noted either following a standard rehabilitation protocol or an enhanced protocol with VR training. The underlying reason might have been that the four-week interval between surgery and assessment was possibly too short to demonstrate a significant clinical advantage of VR-enhanced rehabilitation. We have chosen 4-week interval between assessments because rehabilitation standards of the National Health Service after TKR include three- or four-weeks stationary rehabilitation protocol. Other studies have typically used rehabilitation protocols with at least an eight-week duration [7]. Another reason why the addition of VR-based rehabilitation in the short-term postoperative period may have failed to bring about expected outcomes could be a slow rate of recovery of musculoskeletal function in patients following total knee replacement surgery [32]. The VR group registered nonsignificant decreases across the linear parameters of postural stability in both eyes-open and eyes-closed tests. The eyes-open test additionally showed a significantly smaller elliptical area and range of sway in the sagittal plane, which suggests that a longer course of VR-enhanced rehabilitation could possibly lead to significant improvements in postural stability. Importantly, the standard rehabilitation group registered a nonsignificant increase in the values of the linear parameters assessed, suggesting poorer stability. However, a comparison of the two groups failed to reveal any significant differences. As stated above, the linear measures of postural stability used in this study did not demonstrate significant benefits from the use of VR post-TKR at this stage of ongoing rehabilitation.

Therefore, the present authors decided to use sample entropy as a more sophisticated research tool. The entropy family quantifies signal regularity [33]. A more regular CoP pattern indicates that the postural behavior is more rigid [15]. A decrease in complexity is related to functional decline and a more rigid postural behavior in dysfunctional balance control during perturbations [34]. Unfortunately, the use of a nonlinear parameter did not bring about the expected results: no statistically significant differences were detected. In addition, regression analysis revealed that sample entropy depended on sex (with greater entropy in males), body weight, visual feedback (eyes open vs. eyes closed, with greater entropy registered in the eyes-closed test) and age. These results have been corroborated by other papers [35,36]. A literature review [37] found that across all studies, the absence of vision led to a decrease in sample entropy values compared to when the eyes were open. Moreover, sample entropy was lower for older adults than for young people [38,39]. In the present study, the lack of visual feedback caused slight increases in sample entropy with a significant increase in the coronal plane, suggesting less regular sway. It may thus be hypothesized that such experimental conditions encourage the use of the body’s automatic balance mechanisms [15]. Standing with eyes closed (i.e., creating an internal focus by increasing task difficulty through visual deprivation), causes that more information about the quality of somatosensory and vestibular feedback are given than during standing with eyes open. The nonlinear system approach helps evaluate the temporal component of the movement variation in CoP with regards to how motor behavior develops over time. The regularity measure (entropy family) are sensitive to the visual feedback removal. Donker, Roerdink, Greven and Beek [15] proofed that standing with eyes closed significantly increased CoP regularity (indexed by a decrease in SampEn). While, withdrawing attention from postural control (i.e., performing a cognitive dual task while standing with eyes closed) led to greater irregularity (increase of SampEn) and smaller variability, suggesting an increase in the ‘‘efficiency”, or ‘‘automaticity’’ of postural control. Therefore, it may also be hypothesized that balance training with eyes closed will generate greater improvements in balance by maximizing the effort of the other (nonvisual) systems responsible for body balance than exercises with eyes open or with visual biofeedback. However, this notion should be investigated in a separate study.

In summary, four weeks after TKR appears to be too early for an assessment of postural stability to produce coherent findings. This period was too short to obtain a significant improvement in balance, or such improvements can become visible over time. The sample entropy data indicate that the complexity of the body’s balance mechanisms did not improve.

## 5. Limitation of the Study

The presented study has some limitations, what include: small number of participants. Statistical power was in range 0.0561 (for SampEn_ML during trial with eyes closed) up to 0.7901 (for SampEn_AP during trial with eyes open). Thus, required number of participants should be much higher than 21 persons.

## Figures and Tables

**Table 1:** Characteristics of the participants (mean ± SD).

Group	Age (years)	Body Mass (kg)	Body Height (cm)	Body Mass Index BMI (kg/m^2^)
Study group VR (n = 21)	69 ± 4.76	84 ± 14.32	166 ± 10.03	31 ± 3.33
Control group C (n = 21)	68 ± 7.73	87 ± 17.75	168 ± 13.08	31 ± 4.42

**Table 2:** Means and standard deviations (SD), 95% confidence interval (CI) for means of linear and nonlinear parameters for pre- and post-rehabilitation results in bipedal standing with and without visual feedback, where * indicates significant differences, *p* < 0.05; ↑ indicates an increase in a parameter value after rehabilitation and ↓ indicates a decrease.

	Study Group			Control Group			Study vs. Control	
Parameters	Before (Mean ± SD, 95% CI)	*p*-Value and Relation	After (Mean ± SD, 95% CI)	Before (Mean ± SD, 95% CI)	*p*-Value and Relation	After (Mean ± SD, 95% CI)	*p*-Value and Relation for Before	*p*-Value and Relation for After
	Eyes Open							
SampEn CoP_ML	0.29 ± 0.16 (0.22; 0.36)	*p* = 0.2891 ↓	0.24 ± 0.13 (0.18; 0.30)	0.23 ± 0.11 (0.18; 0.28)	*p* = 0.1492 ↑	0.26 ± 0.14 (0.20; 0.32)	*p* = 0.2371	*p* = 0.6506
SampEn CoP_AP	0.2 ± 0.06 (0.17; 0.23)	*p* = 0.1924 ↑	0.24 ± 0.12 (0.18; 0.30)	0.27 ± 0.1 (0.22; 0.31)	*p* = 0.1396 ↓	0.24 ± 0.12 (0.18; 0.29)	*p* = 0.0305	*p* = 0.9198
Range_ML [cm]	1.34 ± 0.58 (1.08; 1.60)	*p* = 0.0929 ↓	1.12 ± 0.41 (0.93; 1.31)	1.28 ± 0.53 (1.03; 1.52)	*p* = 0.7676 ↑	1.3 ± 0.63 (1.02; 1.59)	*p* = 0.6689	*p* = 0.3023
Range_AP [cm]	2.61 ± 0.91 (2.20; 3.02)	*p* = 0.0354 ↓*	2.13 ± 0.74 (1.80; 2.47)	2.41 ± 0.69 (2.10; 2.73)	*p* = 0.7281 ↑	2.5 ± 0.96 (2.06; 2.93)	*p* = 0.7341	*p* = 0.2272
Path Length [cm]	45.43 ± 11.68 (40.11; 50.75)	*p* = 0.0581 ↓	39.82 ± 8.54 (35.93; 43.71)	49.07 ± 13.97 (42.71; 55.43)	*p* = 0.7676 ↑	51.88 ± 23.37 (41.23; 62.51)	*p* = 0.5628	*p* = 0.1824
CoP mean velocity [cm/s]	1.52 ± 0.39 (1.33; 1.69)	*p* = 0.0605 ↓	1.33 ± 0.28 (1.20; 1.46)	1.64 ± 0.47 (1.42; 1.85)	*p* = 0.7412 ↑	1.73 ± 0.78 (1.37; 2.08)	*p* = 0.5799	*p* = 0.1703
Ellipse Area [cm^2^]	2.47 ± 2.06 (1.53; 3.41)	*p* = 0.0228 ↓*	1.65 ± 0.9 (1.24; 2.05)	2.07 ± 1.15 (1.54; 2.59)	*p* = 0.3218 ↑	2.4 ± 1.7 (1.62; 3.17)	*p* = 0.8800	*p* = 0.2177
	**Eyes Closed**							
SampEn CoP_ML	0.3 ± 0.17 (0.22; 0.37)	*p* = 0.4979 ↓	0.28 ± 0.14 (0.21; 0.34)	0.29 ± 0.13 (0.22; 0.35)	*p* = 0.0792 ↓	0.24 ± 0.15 (0.17; 0.31)	*p* = 0.8602	*p* = 0.2371
SampEn CoP_AP	0.27 ± 0.09 (0.22; 0.31)	*p* = 0.5663 ↑	0.28 ± 0.11 (0.23; 0.33)	0.31 ± 0.13 (0.25; 0.37)	*p* = 0.9861 ↑	0.31 ± 0.15 (0.24; 0.37)	*p* = 0.3651	*p* = 0.8405
Range_ML [cm]	1.5 ± 0.91 (1.08; 1.91)	*p* = 0.6389 ↓	1.25 ± 0.41 (1.06; 1.44)	1.7 ± 1.56 (0.99; 2.41)	*p* = 0.3134 ↑	2.12 ± 1.92 (1.25; 2.99)	*p* = 0.8503	*p* = 0.2523
Range_AP [cm]	2.85 ± 0.97 (2.41; 3.29)	*p* = 0.6813 ↓	2.7 ± 0.86 (2.31; 3.09)	2.83 ± 0.91 (2.42; 3.25)	*p* = 0.6021 ↑	3.18 ± 1.46 (2.52; 3.85)	*p* = 0.9598	*p* = 0.3265
Path Length [cm]	57.28 ± 15.45 (50.25; 64.32)	*p* = 0.7676 ↓	54.78 ± 19.38 (45.95; 63.60)	65.94 ± 28.46 (52.98; 78.90)	*p* = 0.3391 ↑	76.52 ± 45.63 (55.75; 97.29)	*p* = 0.5460	*p* = 0.0871
CoP mean velocity [cm/s]	1.91 ± 0.51 (1.68; 2.14)	*p* = 0.7676 ↓	1.82 ± 0.65 (1.53; 2.12)	2.19 ± 0.95 (1.76; 2.62)	*p* = 0.2958 ↑	2.55 ± 1.52 (1.86; 3.24)	*p* = 0.6060	*p* = 0.0943
Ellipse Area [cm^2^]	98.48 ± 438.45 (-101.1; 298.06)	*p* = 0.1305 ↓	2.27 ± 1.18 (1.73; 2.80)	2.94 ± 2.18 (1.95; 3.93)	*p* = 0.1541 ↑	5.15 ± 5.58 (2.61; 7.69)	*p* = 0.7341	*p* = 0.1908
